# Reporter Virus-Based High-Throughput Screening Identifies LM-10 as a Novel Inhibitor Targeting KRM1 for Coxsackievirus A10 Blockade

**DOI:** 10.3390/ijms27219460

**Published:** 2026-10-23

**Authors:** Haimeng Li, Wenrui Gai, Hailan Yao, Yuchao Gu, Yuyang Li, Minlong Chen, Guangyuan Luo, Jianfeng Zhou, Yu Tang, Zhengyang Wang, Dehai Li, Yingzi Cui

**Affiliations:** 1 Key Laboratory of Marine Drugs Ministry of Education, School of Medicine and Pharmacy, Sanya Oceanographic Institution, Ocean University of China, Qingdao 266003, China; lihaimeng1029@163.com (H.L.); wr_gai666@163.com (W.G.); liyuyang59966@163.com (Y.L.); luogy331@stu.ouc.edu.cn (G.L.); jfzhou@ouc.edu.cn (J.Z.); tangyu@ouc.edu.cn (Y.T.); wzy26@stu.ouc.edu.cn (Z.W.); 2 Capital Center for Children’s Health, Capital Medical University, Capital Institute of Pediatrics, Beijing 100020, China; helen919@163.com; 3 Qingdao Center of Technology Innovation for Shark Antibody Development, College of Biological Engineering, Qingdao University of Science and Technology, Qingdao 266042, China; guych@qust.edu.cn; 4 CAS Key Laboratory of Pathogen Microbiology and Immunology, Institute of Microbiology, Chinese Academy of Sciences, Beijing 100101, China; chenminlong21@mails.ucas.ac.cn; 5 State Key Laboratory of Biopharmaceutical Preparation and Delivery, Institute of Process Engineering, Chinese Academy of Sciences, Beijing 100190, China

**Keywords:** Coxsackievirus A10, structure-based virtual screening, high-content screening, KRM1, viral entry inhibitors, receptor blockade

## Abstract

Enteroviruses causing hand, foot, and mouth disease, including currently circulating Coxsackievirus A10 (CVA10) and Coxsackievirus A6 (CVA6), rely on KREMEN1 (KRM1) for cell entry, making the virus–KRM1 interface a potential target for antiviral intervention. We screened GLMarNP, an in-house library of about 3000 natural products and derivatives. Candidates targeting the KRM1 ectodomain were selected by blind docking, drug-likeness assessment, and scaffold diversity analysis. To enable high-throughput antiviral screening, we inserted eGFP into a full-length infectious CVA10 cDNA clone to generate a reporter virus designated eGFP-A10. The virus was rescued in HEK293T cells and produced robust fluorescence in RD cells over serial passages. Among 29 candidate compounds, LM-10 showed the strongest antiviral activity and exhibited low cytotoxicity. Mechanistically, LM-10 bound KRM1 and acted early in the viral life cycle. Molecular docking suggested that LM-10 binds the KR domain at a site overlapping the VP2 EF-loop contact region. KRM1 also acts as a DKK1 receptor to regulate Wnt/β-catenin signaling. LM-10 did not significantly affect this pathway in the TOP-GFP reporter assay or in transgenic zebrafish embryos. These findings establish a practical CVA10 reporter-virus platform and support steric blockade of the receptor interface as a potential antiviral strategy.

## 1. Introduction

Hand, foot, and mouth disease (HFMD) is a common pediatric infectious disease caused by multiple enteroviruses. Coxsackievirus A10 (CVA10) has emerged as an increasingly important etiological agent of HFMD [1]. Although most CVA10 infections are self-limiting, severe cases may involve cardiopulmonary and neurological complications and can be fatal [2]. Currently, no vaccines or antiviral therapies are approved specifically for CVA10. Previous anti-enterovirus drug development has largely focused on viral capsid proteins [3], but clinical translation has been limited by narrow antiviral coverage [4,5], safety concerns [6,7], and insufficient efficacy [8]. These limitations highlight the need for alternative antiviral strategies.

Targeting genetically stable cellular entry receptors may complement conventional antiviral strategies by raising the barrier to viral escape and providing broad, durable activity against rapidly evolving RNA viruses that share the same receptor [9,10]. Kringle-containing transmembrane protein 1 (KRM1) has been identified as a critical entry receptor for a major group of HFMD-associated enteroviruses, including the widely circulating CVA6 [11], along with CVA2, CVA3, CVA4, CVA5, CVA8, CVA10, and CVA12 [12,13]. This shared receptor usage positions KRM1 as a promising target for broad-spectrum intervention against HFMD-associated enteroviruses. Moreover, the structural basis for enterovirus recognition by KRM1 has been defined. The KRM1 ectodomain comprises KR, WSC, and CUB domains [14], with the KR and WSC domains mediating CVA10 attachment and uncoating by engaging the viral capsid above the canyon [15]. This defined virus–receptor interface provides a structural basis for screening KRM1-targeted antivirals.

Despite its therapeutic potential, the discovery and validation of KRM1-targeted inhibitors have been hindered in part by the lack of rapid, quantitative, and scalable assays for CVA10 infection. Conventional cytopathic effect (CPE) and plaque assays are time-consuming and poorly suited for high-throughput screening. Reporter viruses provide an efficient alternative for direct and quantitative monitoring of infection in living cells [16,17,18,19]. Meanwhile, because KRM1 serves as a high-affinity DKK receptor that regulates Wnt/β-catenin signaling [20,21], a key consideration in developing KRM1-targeted antivirals is to inhibit viral entry while preserving its physiological signaling functions.

In this study, we virtually screened an in-house library of marine-derived natural products and derivatives against the KRM1 extracellular domain and generated a stable eGFP-expressing CVA10 reporter virus, designated eGFP-A10, for functional screening. This integrated approach identified LM-10, a KRM1-targeting molecule that inhibited infection by KRM1-dependent enteroviruses at an early stage without detectably perturbing Wnt/β-catenin signaling or causing developmental toxicity in zebrafish. Together, our findings establish a fluorescence-based CVA10 screening platform and support KRM1-directed entry blockade as a potential antiviral strategy against HFMD-associated enteroviruses.

## 2. Results

### 2.1. Molecular Properties and Drug-likeness of GLMarNP

**Figure 1:**
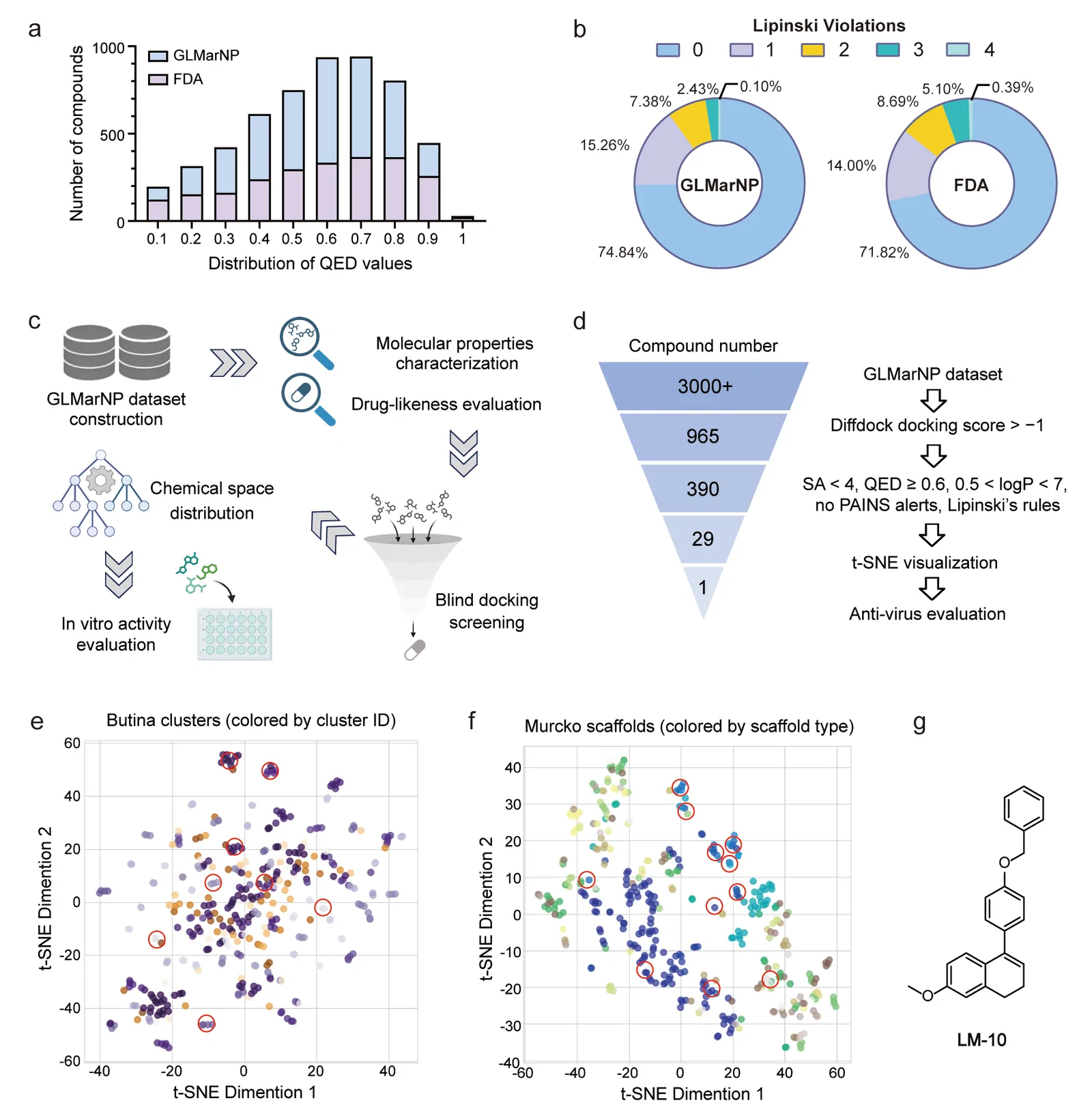
Screening workflow and chemical-space prioritization of candidate natural compounds. (**a**) Drug-likeness (QED) analysis of the GLMarNP and FDA-approved datasets. QED distributions are shown as histograms. (**b**) Distribution of Lipinski’s Rule-of-Five violations in the GLMarNP and FDA-approved datasets. (**c**) Overview of GLMarNP and the screening pipeline. (**d**) KRM1-targeted blind docking and drug-likeness filtering of the GLMarNP dataset yielded 390 compounds for chemical-space analysis. Twenty-nine compounds were subsequently prioritized for in vitro antiviral evaluation, from which LM-10 was selected as the candidate hit. (**e**,**f**) t-distributed stochastic neighbor embedding (t-SNE) visualization of the 390 filtered compounds based on Morgan fingerprints with Butina clustering (**e**) or MACCS keys with Murcko scaffold classification (**f**). Red circles indicate compounds selected for in vitro testing. (**g**) Chemical structure of LM-10.

Natural products provide structurally diverse scaffolds for bioactive compound discovery. We therefore utilized GLMarNP, an existing in-house library comprising more than 3000 marine-derived natural products and derivatives assembled over several decades. Six physicochemical properties were calculated for all compounds in GLMarNP and visualized using violin plots (Figure S1). GLMarNP compounds and FDA-approved drugs showed broadly similar distributions across these properties. Drug-likeness was further evaluated using QED scores, and the results were plotted as stacked bar plots (Figure 1a). Compounds with no more than one Rule-of-Five violation accounted for 85.82% and 90.10% of the FDA-approved drug and GLMarNP datasets, respectively (Figure 1b). Together, these results indicate that GLMarNP occupies chemical space comparable to that of FDA-approved drugs and represents a viable source for virtual screening.

### 2.2. Virtual Screening of KRM1-Targeted Compounds

To identify candidate KRM1-targeted antivirals, we screened GLMarNP against the KRM1 ectodomain (Figure 1c,d). DiffDock-based blind docking [22] identified 965 compounds with confidence scores above −1. These compounds were subsequently filtered using drug-likeness criteria, including a SA score below 4, a QED of at least 0.6, a logP between 0.5 and 7, no PAINS alerts, and compliance with at least three of the four Ro5 criteria. These moderately permissive thresholds were used to accommodate the structural complexity of natural products, yielding 390 candidates.

Structural similarity and chemical-space distribution among these compounds were visualized using t-SNE [23] based on Morgan fingerprints [24] (Figure 1e). Compounds were colored according to Butina clustering [25] defined by Tanimoto similarity, revealing multiple distinct structural clusters. Complementary analyses using MACCS fingerprint [26] and Murcko scaffold [27] revealed substantial diversity in molecular features and core frameworks (Figure 1f). Based on DiffDock confidence scores, drug-like properties, structural diversity, and material availability (>10 mg), 29 compounds representing multiple structural clusters were selected for experimental evaluation.

### 2.3. Construction and Validation of the eGFP-A10 Reporter Virus

To establish a quantitative platform for monitoring CVA10 infection and evaluating antiviral candidates, we generated a full-length reporter virus. CVA10 has an approximately 7.4 kb positive-sense RNA genome containing a single open reading frame flanked by 5′ and 3′ untranslated regions. The encoded polyprotein is processed into the P1, P2, and P3 regions; P1 gives rise to the capsid proteins VP4, VP2, VP3, and VP1, whereas P2 and P3 encode non-structural proteins involved in viral replication [28]. By retaining replication competence and infectious progeny production, the full-length eGFP-A10 reporter virus enables direct and quantitative monitoring of infection and antiviral activity across the complete viral life cycle.

**Figure 2:**
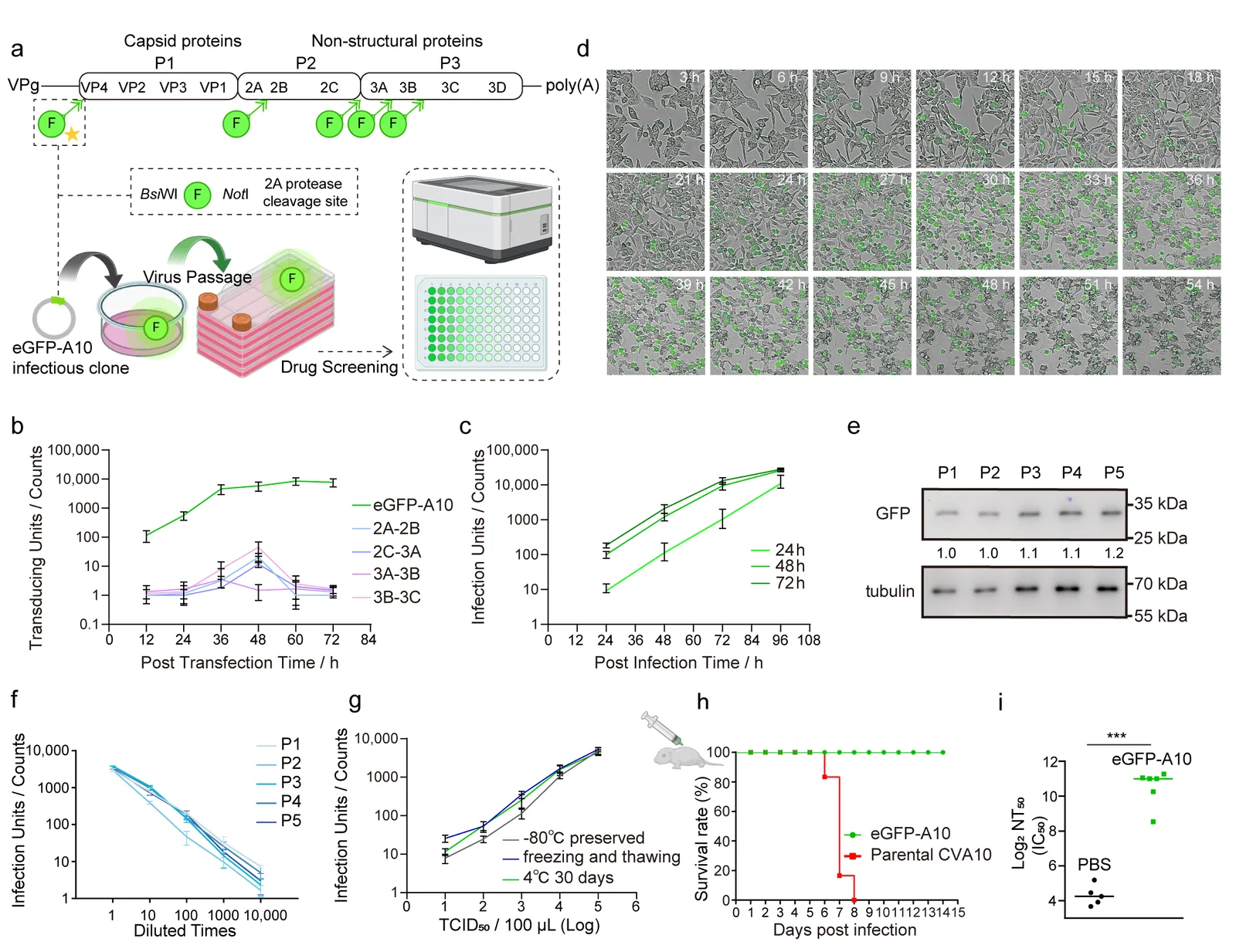
Construction and characterization of the eGFP-A10 reporter virus. (**a**) Design of the eGFP-A10 infectious clone. The eGFP coding sequence followed by a 2A protease cleavage site was inserted into the CVA10 genome at the indicated candidate sites, including the 5′UTR-VP4 junction (indicated by a five-pointed star) and the 2A-2B, 2C-3A, 3A-3B, and 3B-3C junctions. (**b**) Fluorescence-based comparison of reporter constructs following transfection of HEK293T cells. Fluorescence was measured every 12 h using the CQ1 platform. GFP-positive cells were counted, and the data were analyzed using GraphPad Prism 11.1.0 and presented as mean ± SD. (**c**) RD cells were infected with eGFP-A10 preparations harvested at 24 h, 48 h, and 72 h, and GFP signals were monitored at the indicated times post-infection. (**d**) Representative images showing GFP expression and cytopathic changes in eGFP-A10-infected RD cells. Images were acquired hourly using a Revvity high-content imaging system and are shown at 3 h intervals (*n* = 4). (**e**) Western blotting analysis of GFP expression during serial passage of eGFP-A10. RD cells were infected with virus collected at the indicated passages, and cell lysates were harvested for GFP detection by Western blotting. Tubulin served as the loading control. (**f**) Comparison of eGFP-A10 infectivity across passages 1–5 (P1–P5). Virus stocks from each passage were subjected to five consecutive tenfold dilutions and used to infect RD cells. GFP fluorescence was imaged at 24 h post-infection using the CQ1 platform. (**g**) Stability of eGFP-A10 following storage at 4 °C or −80 °C or repeated freeze–thaw cycles over 30 days. Viral infectivity was assessed in RD cells by CQ1 imaging at 24 h post-infection. (**h**) Survival of 7-day-old BALB/c mice inoculated with equivalent titers of eGFP-A10 or parental CVA10. Mice were monitored daily for survival for 14 days (*n* = 6–12 per group). (**i**) At 14 dpi, serum samples were serially diluted, incubated with eGFP-A10, and then applied to RD cells. Infection was quantified using the CQ1 platform, and NT_50_ values were calculated as the reciprocal serum dilution, resulting in a 50% reduction in infection relative to the virus-only control. Each point represents one mouse, and the horizontal line indicates the mean. Statistical significance was determined using two-tailed Student’s *t*-tests: *** *p* < 0.001.

To identify a permissive site for eGFP insertion, we designed five full-length CVA10 constructs based on the infectious cDNA clone (GenBank accession no. MT263729), with eGFP inserted at the VP4 N terminus or at the 2A-2B, 2C-3A, 3A-3B, and 3B-3C junctions (Figure 2a). The inserted eGFP reporter was followed by a viral 2A protease cleavage site (AITTL) to ensure proper separation of eGFP from the viral polyprotein [29,30]. Reporter expression from the five constructs was initially evaluated by monitoring eGFP fluorescence following transfection. The construct carrying eGFP at the N terminus of VP4, designated eGFP-A10, produced markedly stronger fluorescence than the other four constructs (Figure 2b). This finding is consistent with previous reports showing that the 5′ UTR-VP4 junction can accommodate reporter-gene insertion [19] and supported the selection of eGFP-A10 for subsequent virus rescue and characterization.

We next determined the optimal harvest time for progeny virus and characterized the infection kinetics of eGFP-A10 in RD cells. Supernatants collected at 24, 48, and 72 h post-transfection were used to infect fresh RD cells, and eGFP fluorescence was monitored over time. The supernatant collected at 48 h post-transfection produced robust infection and was selected for subsequent experiments (Figure 2c). During infection, GFP fluorescence became detectable at approximately 9 h post-infection and increased markedly by 24 h post-infection (Figure 2d). After approximately 50 h, the fluorescence signal declined concomitantly with cytopathic effects, likely owing to the loss of infected cells (Figure 2d). These kinetics indicate that eGFP-A10 enables time-resolved visualization of CVA10 infection in living cells.

Because inserted reporter genes can be lost during viral propagation through deletion or recombination [16], we next examined the stability of reporter expression during serial passage. eGFP-A10 was serially passaged in HEK293T cells, and viruses collected from each passage were subsequently used to infect RD cells. RD cells infected with virus from each passage showed detectable GFP protein expression (Figure 2e) and fluorescence signals (Figure 2f). In addition, sequencing of eGFP-A10 at passage P7 detected no amino acid mutations in the insertion region (Figure S2), further confirming the genetic stability of the reporter virus during serial passage. We also assessed the stability of eGFP-A10 under different storage conditions. eGFP-A10 stocks stored at 4 °C or −80 °C, or subjected to daily freeze–thaw cycles for one month, retained comparable infectivity in RD cells (Figure 2g). These results demonstrate that eGFP-A10 retains reporter expression and infectivity during serial passage and routine storage.

Furthermore, we evaluated the virulence and immunogenicity of eGFP-A10 in neonatal mice. Compared with the parental CVA10, eGFP-A10 showed markedly reduced lethality. Following intraperitoneal injection of the same viral dose, all mice infected with parental CVA10 died by 8 days post-infection, whereas all eGFP-A10-infected mice survived until the experimental endpoint (Figure 2h). Despite its reduced virulence, eGFP-A10 induced detectable serum neutralizing antibody titers at 14 days post-infection (Figure 2i). These results demonstrate that eGFP-A10 exhibits attenuated virulence while retaining immunogenicity in neonatal mice. Together, these properties support its use for quantitative monitoring of CVA10 infection and antiviral screening.

### 2.4. LM-10 Blocks KRM1-Dependent Enterovirus Infection

**Figure 3:**
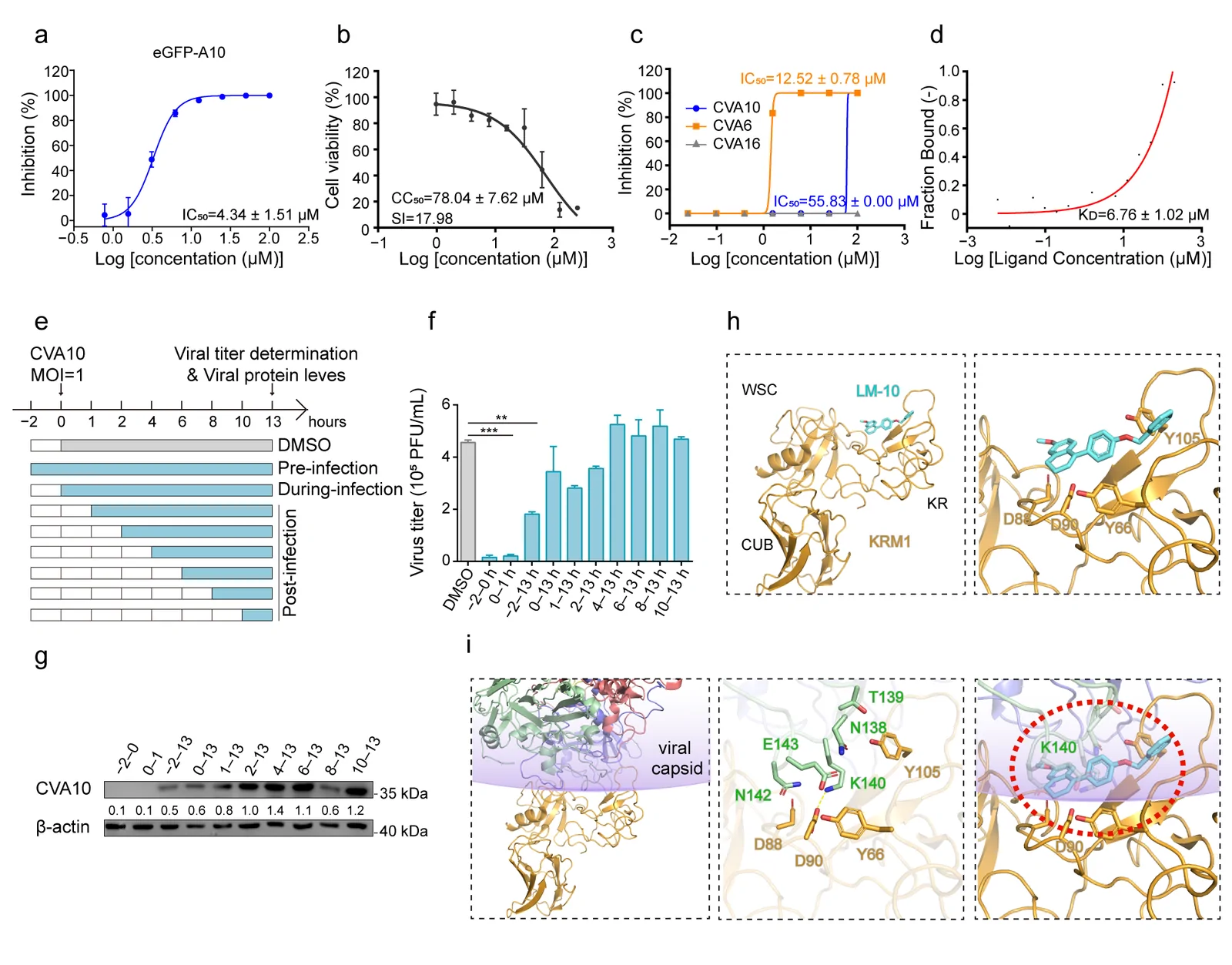
LM-10 inhibits KRM1-dependent CVA10 entry. (**a**) Antiviral activity (IC_50_) of LM-10 against eGFP-A10, quantified using a fluorescence-based infection assay. LM-10 was twofold serially diluted from 100 μM, incubated with 10^4^ TCID_50_ of eGFP-A10 at 37 °C for 1 h, and then added to RD cells. GFP fluorescence was measured at 24 h post-infection to determine the IC_50_. IC_50_ values are presented as mean ± SD from three independent experiments, each performed in triplicate. (**b**) Cytotoxicity of LM-10 determined using a CCK-8 assay. RD cells were treated with LM-10 at concentrations serially diluted twofold from a starting concentration of 250 μM (*n* = 5). Following 24 h of treatment, CCK-8 reagent (10 μL) was added, followed by incubation at 37 °C for 90 min. Cell viability was determined by measuring absorbance at 450 nm (OD_450_). (**c**) Inhibition of CVA10, CVA6, and CVA16 infection by LM-10 in a CPE assay. LM-10 was twofold serially diluted from 100 μM (seven concentrations; six replicates per concentration). Cells were preincubated with the indicated LM-10 concentrations at 4 °C for 30 min and then infected with 100 TCID_50_ of virus at 37 °C for 3 days. CPE was recorded to calculate the IC_50_. Data are presented as mean ± SD. (**d**) Microscale thermophoresis (MST) analysis of LM-10 binding to labeled KRM1. LM-10 was twofold serially diluted starting from 200 μM and mixed with labeled KRM1 at a final concentration of 100 nM. A 10 mM phosphate buffer, pH 7.4 containing 137 mM NaCl, 2.7 mM KCl and 0.05% Tween-20 was used. Binding curves were generated using a Monolith instrument, and the K_D_ value was determined by curve fitting, with an S/N > 5 for all measurements. Fraction bound is plotted against ligand concentration. Data are representative of three independent experiments. All measurements were performed on a Monolith instrument at low MST power and 20% excitation power. (e-g) Time-of-addition analysis of LM-10 during CVA10 infection. RD cells were infected with CVA10 at an MOI of 1, and LM-10 (50 μM) or DMSO was added before infection (−2 h), during infection (0 h), or at the indicated times after infection (1–10 h) (**e**). Viral titers in supernatants collected at 13 h post-infection were determined by plaque assay (**f**), and intracellular CVA10 2C protein levels were analyzed by Western blotting (**g**). Statistical significance was determined using two-tailed Student’s *t*-tests: ** *p* < 0.01 and *** *p* < 0.001. (**h**,**i**) DiffDock-predicted KRM1–LM-10 complex and structural model of viral binding blockade. The CVA10–KRM1 complex model shows that VP2 residues N138-E143 contact KRM1 residues Y66, D88, D90, and Y105. VP2 K140 forms hydrogen bonds with KRM1 D88 and D90, while the predicted LM-10 binding pose sterically occludes this VP2-binding site. VP1, VP2, VP3, and KRM1 are shown in blue, green, red, and orange, respectively; LM-10 is shown in cyan. The key interacting residues are displayed as sticks and colored by element. Hydrogen bonds are represented by dashed lines.

We next used eGFP-A10 to evaluate the antiviral activity of compounds prioritized by virtual screening. Encouragingly, all twenty-nine representative hit compounds exhibited moderate to good antiviral activity, with nine showing IC_50_ values below 20 μM (Table 1 and Figure S3). Among them, LM-10 (chemical structure shown in Figure 1g) showed the highest inhibitory activity, with an IC_50_ of 4.34 μM (Figure 3a). CCK-8 analysis showed that LM-10 had a CC_50_ of 78.04 μM in RD cells (Figure 3b), corresponding to a selectivity index of 17.98 (SI = CC_50_/IC_50_). This separation between antiviral potency and cytotoxicity indicated that the inhibitory effect was unlikely to result from general cytotoxicity. LM-10 was therefore selected for further characterization.

We next evaluated the antiviral activity of LM-10 against wild-type (WT) enteroviruses using a CPE-based assay. LM-10 inhibited wild-type CVA10 and CVA6 infection, with IC_50_ values of 55.83 and 12.52 μM, respectively (Figure 3c). The higher IC_50_ against WT CVA10 than against eGFP-A10 may partly reflect differences in assay endpoints, with fluorescence measured at 24 h and CPE assessed at 72 h post-infection. In contrast, LM-10 showed no detectable activity against CVA16, which does not depend on KRM1 for entry (Figure 3c). Given that both CVA10 and CVA6, but not CVA16, use KRM1 as an entry receptor, this receptor-dependent antiviral profile supports KRM1 as a candidate cellular target of LM-10. Microscale thermophoresis (MST) further demonstrated concentration-dependent binding of LM-10 to KRM1, with a dissociation constant (K_D_) of 6.76 μM (Figure 3d), confirming their direct interaction.

We then performed a time-of-addition assay to define the stage of the CVA10 life cycle affected by LM-10 (Figure 3e). RD cells were infected with CVA10 at 0 h, and LM-10 was administered at the indicated time points before, during, or after infection. LM-10 treatment beginning 2 h before infection markedly reduced both progeny virus titers in the supernatant (Figure 3f) and intracellular viral protein levels (Figure 3g), indicating that LM-10 acts predominantly at an early stage of CVA10 infection.

We next used DiffDock to predict the binding mode of LM-10 to KRM1. The top-ranked pose placed LM-10 within the KR-domain groove adjacent to KRM1 D90, a residue critical for CVA10 entry that interacts with the conserved CVA10 VP2 residue K140 [13,15] (Figure 3h). MST analysis showed that two single-point mutations, D88A and D90A, reduced LM-10 binding affinity, with K_D_ values increasing from 6.76 µM for WT KRM1 to 93.71 µM and 33.04 µM for D88A and D90A, respectively (Figure S4). This result further supports the predicted binding region (Figure 3i). Together, these findings suggest that LM-10 may interfere with viral engagement of KRM1 through steric hindrance.

### 2.5. LM-10 Blocks KRM1-Dependent Enterovirus Infection While Preserving Wnt/β-Catenin Signaling

**Figure 4:**
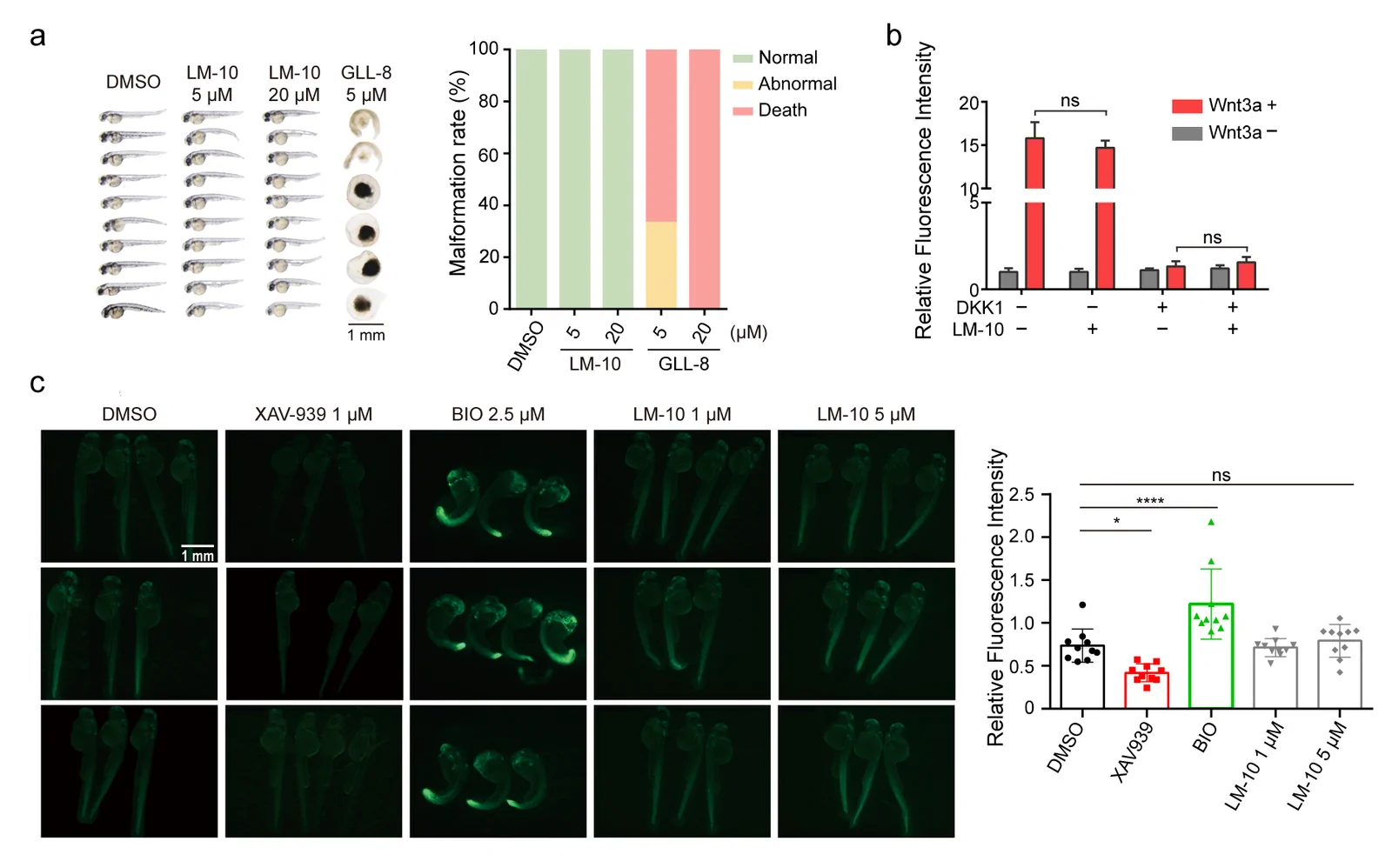
Evaluation of LM-10 developmental toxicity and its effects on Wnt/β-catenin signaling in cultured cells and zebrafish embryos. (**a**) Zebrafish embryo toxicity assay following 48 h exposure to LM-10 or GLL-8, with representative images and quantification of developmental outcomes, including malformation and mortality rates. Both compounds were tested at 5 and 20 μM (*n* = 30). Scale bar, 1 mm. (**b**) TOP-GFP reporter assay evaluating the effect of LM-10 on Wnt/β-catenin signaling in HEK293T cells stimulated with Wnt3a in the presence or absence of DKK1 (1 μg/mL) or LM-10 (20 μM) (*n* = 5). (**c**) Wnt reporter zebrafish embryos treated with the inhibitor XAV-939 (1 μM), the activator BIO (2.5 μM), or LM-10 (1 or 5 μM) for 48 h. Whole-embryo fluorescence intensity was quantified after background subtraction (*n* = 10). Scale bar, 1 mm. Data are presented as mean ± SD. Statistical significance was determined using two-tailed Student’s *t*-tests: * *p* < 0.05, **** *p* < 0.0001; ns, not significant.

A key consideration for receptor-blockade strategies is whether they interfere with receptor-associated physiological signaling [31]. As KRM1 serves as a DKK1 receptor in Wnt/β-catenin signaling [20], we assessed whether LM-10 perturbs this pathway. Given the essential role of Wnt/β-catenin signaling in embryonic development, we first evaluated the developmental toxicity of LM-10 in zebrafish embryos, a sensitive in vivo model for detecting developmental and morphological abnormalities [32,33]. LM-10 caused no overt phenotypic or morphological abnormalities in zebrafish embryos, whereas another candidate, GLL-8, resulted in 66.7% embryo mortality (Figure 4a), indicating no detectable developmental toxicity under the conditions tested.

We next assessed the effects of LM-10 on Wnt/β-catenin signaling in cultured cells and zebrafish embryos. In the Wnt3a-stimulated TOP-GFP reporter assay, LM-10 did not affect Wnt3a-induced reporter activity or DKK1-mediated suppression of this activity (Figure 4b). In Wnt reporter zebrafish embryos, XAV-939 reduced reporter fluorescence, whereas BIO increased fluorescence and induced the characteristic eyeless phenotype. By contrast, LM-10 did not significantly affect reporter fluorescence relative to the DMSO control (Figure 4c). Together, these results indicate that LM-10 does not detectably perturb KRM1-associated Wnt/β-catenin signaling in cultured cells or zebrafish embryos.

## 3. Discussion

Most enterovirus antiviral strategies target viral components, particularly capsid proteins, but their effectiveness may be compromised by viral genetic variation and the emergence of resistant variants [3,34,35,36,37]. Targeting a host entry receptor offers a complementary strategy and may provide broader antiviral coverage and a higher barrier to resistance, particularly when the receptor is shared by multiple viruses. KRM1 is an attractive target because it is used by multiple HFMD-associated EV-A viruses and mediates both attachment and uncoating during CVA10 entry [15].

In this study, we integrated AI-assisted blind docking with an eGFP-A10-based screening platform to identify inhibitors of KRM1-dependent enteroviruses. Blind docking enabled automated prediction of potential ligand-binding sites on KRM1 and virtual screening of our in-house GLMarNP library. We also developed eGFP-A10, a reporter virus that enables direct, quantitative, and high-throughput measurement of CVA10 infection. The reporter facilitated efficient primary screening, with the most promising hits subsequently prioritized for validation against wild-type viruses. Among 29 structurally diverse candidates, LM-10 showed the strongest inhibition of eGFP-A10 infection. Mechanistically, LM-10 directly binds KRM1 and acts at an early stage of infection to inhibit KRM1-dependent enterovirus entry. Molecular docking positioned LM-10 within the KR groove of KRM1, where it sterically blocks binding of the VP2 EF loop to KRM1. Together, these findings support a model in which LM-10 inhibits viral entry by interfering with virus–receptor engagement.

KRM1 is a transmembrane receptor for DKK1 that regulates Wnt/β-catenin signaling, a pathway essential for embryonic development and tissue homeostasis [20]. A central challenge in developing KRM1-directed antivirals is therefore to inhibit viral entry without disrupting receptor-mediated physiological signaling. Although the KR and WSC domains participate in both DKK1 recognition and viral capsid engagement, LM-10 did not alter Wnt3a-induced reporter activity or DKK1-mediated pathway inhibition in cultured cells. LM-10 neither affected Wnt reporter activity nor caused overt developmental abnormalities in zebrafish embryos. These findings support the concept that LM-10 interferes with the virus-contacting surface of KRM1 while preserving its physiological role in Wnt/β-catenin signaling.

This study has several limitations. Although molecular docking, mutagenesis and functional data support interference with KRM1–virus engagement, direct evidence for steric competition with viral particles remains lacking. In addition, co-crystallization of KRM1 with LM-10 did not yield a structure, leaving its precise binding site and mechanism of action unresolved. Similar structural challenges have been reported for ACE2-directed small-molecule inhibitors of SARS-CoV-2 entry [31]. In addition, the therapeutic potential of LM-10 remains to be established in vivo. Although LM-10 showed no significant effects on Wnt signaling or zebrafish development under the tested conditions, further studies are needed to comprehensively evaluate its safety and potential effects on other KRM1-associated physiological functions. Further optimization of its physicochemical and pharmacokinetic properties, together with systematic evaluation of dose, tissue exposure, and route of administration, will be required to achieve effective drug concentrations at sites of viral infection.

An important consideration for receptor-directed antivirals is functional selectivity, because viral receptors are endogenous proteins that participate in signaling, trafficking, enzymatic, and homeostatic processes. Candidate inhibitors should ideally target virus-contacting surfaces that are functionally separable from physiological interfaces, and their safety evaluation should extend beyond general cytotoxicity to receptor-specific functions in relevant tissues and over clinically relevant exposure periods [38]. In the case of KRM1, Wnt/β-catenin regulation represents only one aspect of its biological activity. KRM1 can also function as a dependence receptor that regulates cell survival independently of canonical Wnt signaling: unliganded KRM1 induces cell death, whereas DKK1 binding or heterodimerization with KRM2 suppresses this activity [39,40]. This Wnt-independent activity has been linked to a placental-mammal-specific intracellular motif, underscoring the importance of evaluating receptor-directed candidates in mammalian systems in addition to zebrafish reporter models [39]. Such evaluation should encompass receptor trafficking, oligomerization, partner interactions, Wnt-independent signaling, tissue-specific functions, and the consequences of prolonged exposure. More broadly, incompletely characterized functions of viral receptors may represent latent safety risks that require consideration during receptor-directed antiviral development.

In summary, this study validates KRM1 as a feasible target for antiviral intervention and identifies LM-10 as a lead candidate against KRM1-dependent enteroviruses without detectable disruption of Wnt/β-catenin signaling. It also establishes an integrated framework for the discovery and validation of receptor-directed antivirals.

## 4. Materials and Methods

### 4.1. GLMarNP Assembly and Molecular Properties Calculation

Compounds isolated or synthesized in-house over several decades were curated, standardized, and encoded as SMILES strings. RDKit was employed to calculate six physicochemical properties of therapeutic interest, namely, molecular weight (MW), calculated octanol/water partition coefficient (clogP), topological polar surface area (TPSA), synthetic accessibility (SA), number of hydrogen-bond acceptors (HBA), and number of hydrogen-bond donors (HBD). Drug-likeness was evaluated using Quantitative Estimate of Drug-likeness (QED) scores, which were derived from MW, cLogP, TPSA, HBD, HBA, rotatable bonds, aromatic rings, and structural alerts, and visualized as stacked bar plots. The Lipinski’s Rule-of-Five (Ro5) was applied to evaluate rule violations for each compound in GLMarNP, and the results were visualized as pie charts. The dataset of FDA-approved drugs [41] was used as a reference, and the same calculations were applied to its compounds.

### 4.2. Virtual Screening of an In-House Compound Library and Molecular Docking Against KRM1

Virtual screening was performed using DiffDock. The KRM1 structure was downloaded from the Protein Data Bank (PDB ID: 7BZT), and prepared by retaining the KRM1 chain and removing other chains and solvent molecules. The compounds were obtained from our in-house library, which has been established over several decades and comprises more than 3000 marine-derived natural products and derivatives. Docking results were reported as confidence scores representing the predicted stability of binding poses, with higher scores indicating greater confidence in stable ligand binding. Compounds scoring higher than −1.0 were selected for further evaluation. These compounds were filtered for drug-likeness using the following criteria: SA < 4, QED ≥ 0.6, 0.5 < logP < 7, no pan-assay interference compounds (PAINS) alerts, and compliance with at least three of the four Rule-of-Five criteria. T-distributed stochastic neighbor embedding (t-SNE) analyses were performed separately based on Morgan and MACCS fingerprints. Butina Clustering and Bemis–Murcko scaffold analysis were also used for visualization. Compounds with an available inventory of >10 mg in the in-house library were prioritized as representative candidates for experimental validation.

### 4.3. Synthesis and Characterization of Compound LM-10

LM-10 was synthesized as previously described and characterized by NMR spectroscopy. All spectral data were consistent with reported values [42]. ^1^H NMR (400 MHz, Chloroform-*d*) *δ* 7.51–7.31 (m, 6H), 7.28 (s, 1H), 7.05–6.91 (m, 3H), 6.77 (d, *J* = 2.6 Hz, 1H), 6.63 (dd, *J* = 8.5, 2.6 Hz, 1H), 5.91 (t, *J* = 4.7 Hz, 1H), 5.09 (s, 2H), 3.80 (s, 3H), 2.81 (t, *J* = 7.9 Hz, 2H), 2.36 (td, *J* = 7.9, 4.8 Hz, 2H). ^13^C NMR (101 MHz, DMSO-*d_6_*) *δ* 158.7, 158.1, 138.8, 138.7, 137.6, 133.2, 129.8, 128.9, 128.3, 128.2, 128.0, 126.5, 124.6, 115.1, 114.2, 111.4, 69.7, 55.5, 28.6, 23.4.

### 4.4. eGFP-A10 Virus Production and Stability Assays

A full-length CVA10 cDNA clone was synthesized based on the viral genomic sequence (GenBank accession no. MT263729). The eGFP coding sequence was inserted at five candidate positions within the viral genome: the N terminus of VP4, the 2A-2B junction, the 2C-3A junction, the 3A-3B junction, and the 3B-3C junction. An AITTL sequence recognized by the viral 2A protease was appended to the C terminus of eGFP to permit cleavage of eGFP from the downstream viral polyprotein.

HEK293T cells seeded in 6-well plates were transfected with each designed plasmid construct. Six replicates were performed for each construct. GFP fluorescence signals were recorded every 12 h post-transfection using the CellVoyager CQ1 platform (Yokogawa). The plasmid construct in which the eGFP gene was inserted immediately upstream of the VP4 start codon was identified as the most efficient virus-producing construct. At 48 h post-transfection, the culture supernatant was collected and centrifuged to remove cell debris. The clarified supernatant was used for eGFP-A10 production in subsequent experiments.

For stability assays, the supernatant was aliquoted into three tubes. Two aliquots were stored at 4 °C and −80 °C for one month, respectively, whereas the third aliquot was subjected to daily freeze–thaw cycles over the same period. All virus samples were serially diluted and used to infect RD cells seeded in 96-well plates. Six replicates were performed for each dilution. At the indicated times post-infection, GFP fluorescence was imaged using the CQ1 platform with excitation and emission wavelengths of 488 and 525 nm, respectively. GFP-positive cells were counted, and the data were analyzed using GraphPad Prism 11.1.0 and presented as mean ± SD.

### 4.5. Serial Passage of eGFP-A10

The virus stock recovered from plasmid-transfected cells was designated passage 1 (P1). For serial passage, HEK293T cells seeded in six-well plates were infected with 20 μL of the P1 virus stock and incubated at 37 °C with 5% CO_2_ for 48 h. The culture supernatant was harvested and centrifuged at 5000 × *g* for 5 min. The clarified supernatant, designated P2, was stored at −80 °C and used for the subsequent passage. This procedure was repeated for four consecutive passages, yielding virus stocks from P1 to P5. RD cells seeded in 96-well plates were infected with each passage stock, and GFP fluorescence was monitored at 24 h post-infection using the CQ1 imaging cytometer.

RD cells infected with virus from each passage were harvested at 36 h post-infection for Western blotting. Cells were harvested and lysed in RIPA buffer (Beyotime, P0013B, Shanghai, China) supplemented with protease inhibitors (Beyotime, P1005, Shanghai, China). Protein samples were resolved by SDS-PAGE and transferred to nitrocellulose (NC) membranes (Merck Millipore, HATF00010, Billerica, MA, USA). The membranes were blocked with 5% skim milk in TBST (20 mM Tris-HCl, pH 7.5, 150 mM NaCl, and 0.05% Tween-20) for 1 h at room temperature, followed by overnight incubation at 4 °C with primary antibodies against GFP (1:1000, Thermo Fisher, MA5-15256, Waltham, MA, USA) and tubulin (1:5000, Thermo Fisher, MA5-16308, Waltham, MA, USA). The membranes were washed three times with TBST and then incubated with HRP-conjugated anti-mouse secondary antibodies (1:5000, EASYBIO, BE0102, Beijing, China) for 1 h at room temperature. The membranes were developed with enhanced chemiluminescence (ECL) substrate for 1 min, and signals were captured using a Bio-Rad ChemiDoc (Hercules, CA, USA) imaging system. Band intensities were quantified using ImageJ.JS 1.54t and normalized to the corresponding tubulin signals. The eGFP-A10 virus at passage P7 was subjected to next-generation sequencing, and the resulting sequence alignment was visualized using ESPript 3.2.

### 4.6. In Vivo Pathogenicity of eGFP-A10

All animal experiments were performed according to the protocols approved by the Animal Use and Care Committee of the Institute of Biophysics, Chinese Academy of Sciences (ABSL-2-2024052). Groups of 7-day-old suckling BALB/c mice were inoculated intraperitoneally with CVA10 or eGFP-A10 at a dose of 10^5^ TCID_50_ per mouse (*n* = 6–12 per group). All mice were monitored daily for clinical signs and mortality until 14 days post-infection (dpi). At 14 dpi, serum samples were collected from the mice. Serum samples were serially diluted, incubated with eGFP-A10, and then applied to RD cells. At 24 h post-infection, GFP-positive cells were quantified using the CQ1 platform. Neutralization was calculated relative to the virus-only control, and the 50% neutralization titer (NT_50_) was defined as the reciprocal serum dilution that reduced the number of GFP-positive cells by 50%. NT_50_ values were calculated by nonlinear regression using GraphPad Prism.

### 4.7. eGFP-A10-Based Antiviral Activity Assay

All compounds were dissolved in dimethyl sulfoxide (DMSO) and subsequently subjected to twofold serial dilution in DMEM, starting from a concentration of 100 μM (*n* = 3). RD cells were seeded into 96-well plates at 80% confluence and cultured for 12 h at 37 °C with 5% CO_2_. The tested compounds or DMSO were mixed 1:1 with eGFP-A10 (10^4^ TCID_50_) and incubated at 37 °C for 1 h before infection of RD cells. At 24 h post-infection, GFP fluorescence was imaged using the CQ1 platform. GFP-positive cell counts were quantified. Values were normalized to those of the untreated virus-infected control, and IC_50_ values were calculated by nonlinear regression using GraphPad Prism.

### 4.8. Cytopathic Effect (CPE) Assay

The CVA10 HB09-035 strain (GenBank accession no. MT263729) and the CVA6 HeB081 strain were isolated from Hebei Province. The CVA16 shzh05-1 strain (GenBank accession no. EU262658.1) was isolated from Shenzhen Province. RD cells seeded in 96-well plates 12 h earlier were treated with seven twofold serial dilutions of LM-10 starting at 100 μM and incubated at 4 °C for 30 min (*n* = 6). Subsequently, 100 TCID_50_ of virus was added to the cells, followed by incubation at 37 °C for 3 days. CPE was recorded to determine the IC_50_ of LM-10.

### 4.9. Microscale Thermophoresis (MST)

KRM1 and its mutants (D88A and D90A) were expressed and purified as previously described [15] and labeled using the Monolith^TM^ RED-NHS Labeling Kit (MO-L011, NanoTemper, Munich, Germany) according to the manufacturer’s instructions. LM-10 was prepared as a 16-point twofold serial dilution starting at 200 μM and mixed with labeled KRM1 at a final concentration of 100 nM. The experiment was performed in triplicate. LM-10 was prepared as a 16-point twofold serial dilution starting at 100 μM and mixed with labeled mutant protein at a final concentration of 100 nM. The experiment was performed in duplicate. A 10 mM phosphate buffer, pH 7.4 containing 137 mM NaCl, 2.7 mM KCl and 0.05% Tween-20 was used. After centrifugation at 12,000 rpm for 10 min at 4 °C, the supernatants were loaded into capillaries (MO-K022, NanoTemper, Munich, Germany) and binding affinity was measured on a Monolith instrument (NanoTemper Technologies, Munich, Germany) at low MST power and 20% excitation power. All MST measurements yielded a signal-to-noise ratio (S/N) > 5, ensuring the reliability and reproducibility of the binding data. The MST signal was defined as the ratio of normalized fluorescence intensity (F_norm) before and after heating, and data were processed using the instrument and displayed as Fraction Bound, thereby eliminating differences in absolute signal amplitude between independent experiments and allowing direct comparison of binding curves across conditions. Binding curves were fitted using the K_D_ model in the MO.Affinity Analysis software v2.3 (NanoTemper, Munich, Germany) to obtain dissociation constants (K_D_).

### 4.10. Time of Addition Assay

RD cells were seeded in 12-well plates 1 day before infection with WT CVA10 (HB09-035 strain) at an MOI of 1. LM-10 (50 μM) was added at the indicated times relative to infection: −2, 0, 1, 2, 4, 6, 8, and 10 h [43]. DMSO-treated cells served as the vehicle control. At 13 h post-infection, culture supernatants were collected for viral titration by plaque assay and cell lysates were harvested for Western blot analysis. CVA10 2C and β-actin were detected using mouse monoclonal antibody L8F12 and an anti-β-actin monoclonal antibody (1:3000; TransGen Biotech, HC201, Beijing, China), respectively, followed by HRP-conjugated anti-mouse IgG (1:5000; EASYBIO, BE0102, Beijing, China).

### 4.11. Plaque Assay

RD cells were seeded in 12-well plates and incubated overnight under standard conditions at 37 °C with 5% CO_2_. Supernatants collected from the time-of-addition assay were serially diluted 1:100, 1:1,000, and 1:10,000, and 800 μL of each dilution was added to RD cells in duplicate. After incubation for 1 h, the inoculum was gently removed, and the cells were washed twice with PBS. An equal volume of 2% carboxymethylcellulose was mixed with 2 × DMEM to prepare the overlay medium, which was added at 1 mL per well. After incubation for 48 h, the cells were fixed with 4% formaldehyde for 1 h at room temperature, washed three times with distilled water, and stained with 1 mL of 1% crystal violet for 1 h. Plaques were counted, and viral titers were calculated and expressed as plaque-forming units per milliliter (PFU/mL).

### 4.12. Cytotoxicity Assays

RD cells were treated with twofold serial dilutions of LM-10 starting at 250 μM (*n* = 5). At 24 h after treatment, 10 μL of CCK-8 reagent (PF00004, Proteintech, Rosemont, IL, USA) was added to each well, followed by incubation at 37 °C for 90 min. Absorbance at 450 nm was measured using a multimode microplate reader (Thermo Fisher Scientific, Waltham, MA, USA). Cell viability was normalized to that of the vehicle-treated control, and CC_50_ values were calculated by nonlinear regression using GraphPad Prism.

### 4.13. Zebrafish Strains

All animal procedures were conducted according to the guidelines established by the University Committee on the Use and Care of Animals at the Ocean University of China (OUC-SMP-2026-09-15). Wild-type zebrafish (Danio rerio; Tübingen strain) were used to evaluate LM-10 toxicity, and the Wnt reporter line Tg (7×TCF-Xla.Siam:GFP) was used to assess its effect on Wnt/β-catenin signaling. Zebrafish were maintained at 28.5 °C under a 14 h light/10 h dark cycle and fed twice daily. Embryos were obtained by natural spawning. Adult male and female zebrafish were housed overnight in mating tanks separated by a removable partition. After removal of the partition, embryos were collected within 30 min [44]. Embryos were maintained in embryo medium at 28.5 °C and staged according to standard criteria [45]. At 6 h post-fertilization (hpf), developmentally synchronized, morphologically normal embryos were transferred to 24-well plates at ten embryos per well.

### 4.14. Developmental Toxicity Assessment

Zebrafish embryos were exposed to LM-10 at final concentrations of 5 μM and 20 μM starting at 6 hpf. Control groups were treated with the corresponding vehicle. Separate groups were treated with the hit compounds (IC_50_ < 20 μM) at the same concentrations (5 and 20 μM). Each group included at least three biological replicates. For phenotypic and morphological analyses, embryos were observed and photographed after 48 h of exposure. Survival, malformation, and mortality rates were determined manually [44]. The data were plotted using GraphPad Prism.

### 4.15. TOP-GFP Reporter Assay

The TOP-GFP reporter assay was used to determine whether LM-10 affects the Wnt/β-catenin signaling pathway in addition to blocking CVA10 entry. The TOP-GFP reporter plasmid was kindly provided by Prof. Ye-Guang Chen’s laboratory. HEK293T cells were transfected with the TOP-GFP reporter plasmid and then seeded in 96-well plates. At 24 h post-transfection, the cells were treated with or without Wnt3a-conditioned medium (CM), LM-10 (20 μM), and DKK1 (1 μg/mL) (*n* = 5 per group). At 48 h post-transfection, TCF/LEF transcriptional activity was monitored by imaging GFP fluorescence using the CQ1 platform. The experiment was independently repeated three times. Data were analyzed using GraphPad Prism.

### 4.16. Reporter Assay in Zebrafish Embryos

Tg (7×TCF-Xla.Siam:GFP) zebrafish embryos were exposed from 6 hpf to XAV-939 (1 μM), BIO (2.5 μM), or LM-10 (1 or 5 μM) in E3 embryo medium. Vehicle-treated embryos served as controls [46]. After 48 h of exposure, the embryos were anesthetized with 0.03% tricaine and imaged using a Leica M205 fluorescence stereomicroscope under identical acquisition settings. Whole-embryo regions of interest (ROIs) were manually defined in ImageJ 1.54t, and mean GFP fluorescence intensity was quantified after subtraction of the adjacent background signal. Background-corrected fluorescence intensities were compared among treatment groups and plotted using GraphPad Prism.

### 4.17. Statistical Analysis

Graphical analysis was performed using GraphPad Prism 6. Statistically significant differences were determined using two-tailed Student’s *t*-tests. Statistical significance is indicated in the figures as follows: * *p* < 0.05, ** *p* < 0.01, *** *p* < 0.001, and **** *p* < 0.0001; ns, not significant.

## 5. Conclusions

CV-A10 is one of the major causative agents of hand–foot–mouth disease (HFMD), an acute febrile disease mainly occurring in young children. However, there are no effective vaccines or therapeutic drugs available for CV-A10 infections currently. Targeting the host entry receptor is a promising strategy for blocking viral entry, but a central challenge is to preserve receptor physiology. KREMEN1 (KRM1), a receptor for circulating CVA10 and CVA6 strains that also functions as a DKK receptor in Wnt/β-catenin signaling, provides a representative model for studying receptor-targeted antiviral strategies.

Here, we performed structure-based virtual screening of an in-house natural products and derivatives library and, using an established CVA10 reporter virus screening system, identified LM-10 as a small-molecule inhibitor targeting KRM1. LM-10 bounds directly to KRM1 and selectively blocks KRM1-dependent enterovirus infection. LM-10 was predicted to bind within the KRM1 KR-domain groove, overlapping with the VP2 EF-loop contact hotspot. Importantly, LM-10 did not perturb Wnt3a-induced reporter activity or DKK1-mediated inhibition in vitro. Consistently, LM-10 caused no overt developmental abnormalities in zebrafish embryos and did not significantly interfere with Wnt/β-catenin reporter activity in vivo, highlighting it as a promising antiviral strategy.

## 6. Patents

Y.L., D.L., Y.C., H.L., W.G., Y.T. and J.Z. are named inventors on a patent application related to the work described in this paper. The authors declare no other competing interests.

## Figures and Tables

**Table 1:** DiffDock confidence scores and eGFP-A10-based antiviral IC_50_ values for the 29 candidate compounds.

Compound_ID	IC_50_ (μM)	DiffDock Score	Compound_ID	IC_50_ (μM)	DiffDock Score
LM-10	4.34 ± 1.51	−0.89	TYL-37	25.06 ± 8.07	−0.44
LM-12	12.82 ± 2.37	−0.60	TYL-54	30.93 ± 22.09	−0.57
LM-3	13.81 ± 7.79	−0.91	GLL-1	33.13 ± 2.40	−0.72
LRY-36	14.44 ± 1.00	−0.65	SWC-138	34.17 ± 3.21	−0.63
CJ-9	15.97 ± 5.09	−0.98	GLL-2	34.48 ± 4.20	−0.50
TYL-55	16.15 ± 2.86	−0.29	TYL-72	34.75 ± 16.92	−0.47
LM-1	18.31 ± 3.07	−0.55	TYL-33	35.40 ± 14.30	−0.48
TYL-42	18.68 ± 7.20	−0.74	WZH-194	35.80 ± 20.02	−0.26
GLL-8	19.91 ± 11.09	−0.95	LM-4	37.54 ± 1.68	−0.84
GLL-3	20.49 ± 0.55	−0.90	TYL-32	38.59 ± 11.24	−0.60
LM-2	20.58 ± 6.69	−0.80	GLL-9	41.50 ± 11.33	−0.88
GLL-5	20.94 ± 9.47	−0.63	LM-11	44.66 ± 10.19	−0.21
GLL-6	23.78 ± 6.11	−0.60	LHN-229	56.32 ± 5.56	−0.43
GLL-4	24.28 ± 11.44	−0.57	LHN-5	68.14 ± 21.11	−0.99
LM-6	24.99 ± 6.22	−0.93			

Antiviral efficacy of 29 compounds against eGFP-A10, as determined using a fluorescence-based infection assay. Compounds were twofold serially diluted from 100 μM, incubated with 10^4^ TCID_50_ of eGFP-A10 at 37 °C for 1 h, and then added to RD cells. GFP fluorescence was measured at 24 h post-infection to calculate IC_50_ values. Data are presented as the mean ± SD of three independent experiments, each performed in triplicate.

## Data Availability

All data generated or analysed during this study are included in this published article (and its Supplementary Materials Files).

## Supplementary Materials

The following supporting information can be downloaded at: https://www.mdpi.com/article/10.3390/ijms27219460/s1.
