# Supplementary material for: Reporter Virus-Based High-Throughput Screening Identifies LM-10 as a Novel Inhibitor Targeting KRM1 for Coxsackievirus A10 Blockade
Source: Int J Mol Sci. 2026 Oct 23;27(21):9460. doi: 10.3390/ijms27219460 (PMC13762517; doi:10.3390/ijms27219460)
Supplement: Supplementary file 1 [file ijms-27-09460-s001.zip › ijms-4621608-supplementary.pdf]

Supplementary Materials

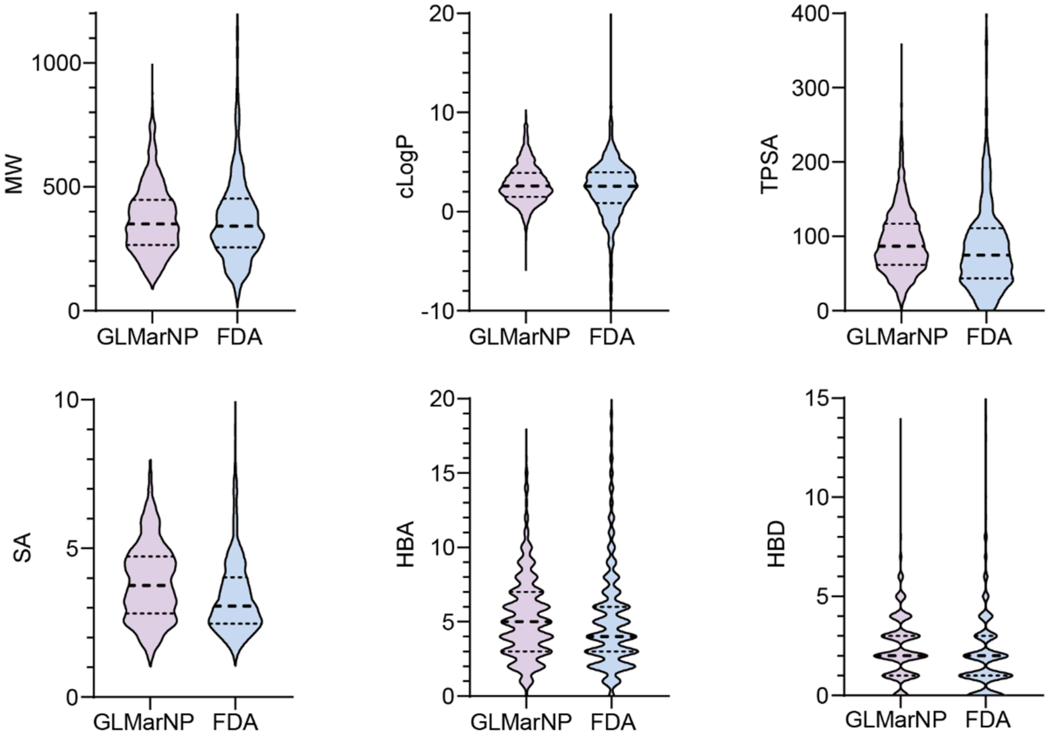

Figure S1. Violin plots of the physicochemical properties in the GLMarNP and FDA-approved datasets. MW, molecular weight; clogP, calculated octanol/water partition coefficient; TPSA, topological polar surface area; SA, synthetic accessibility; HBA, number of hydrogen-bond acceptors; HBD, number of hydrogen-bond donors.

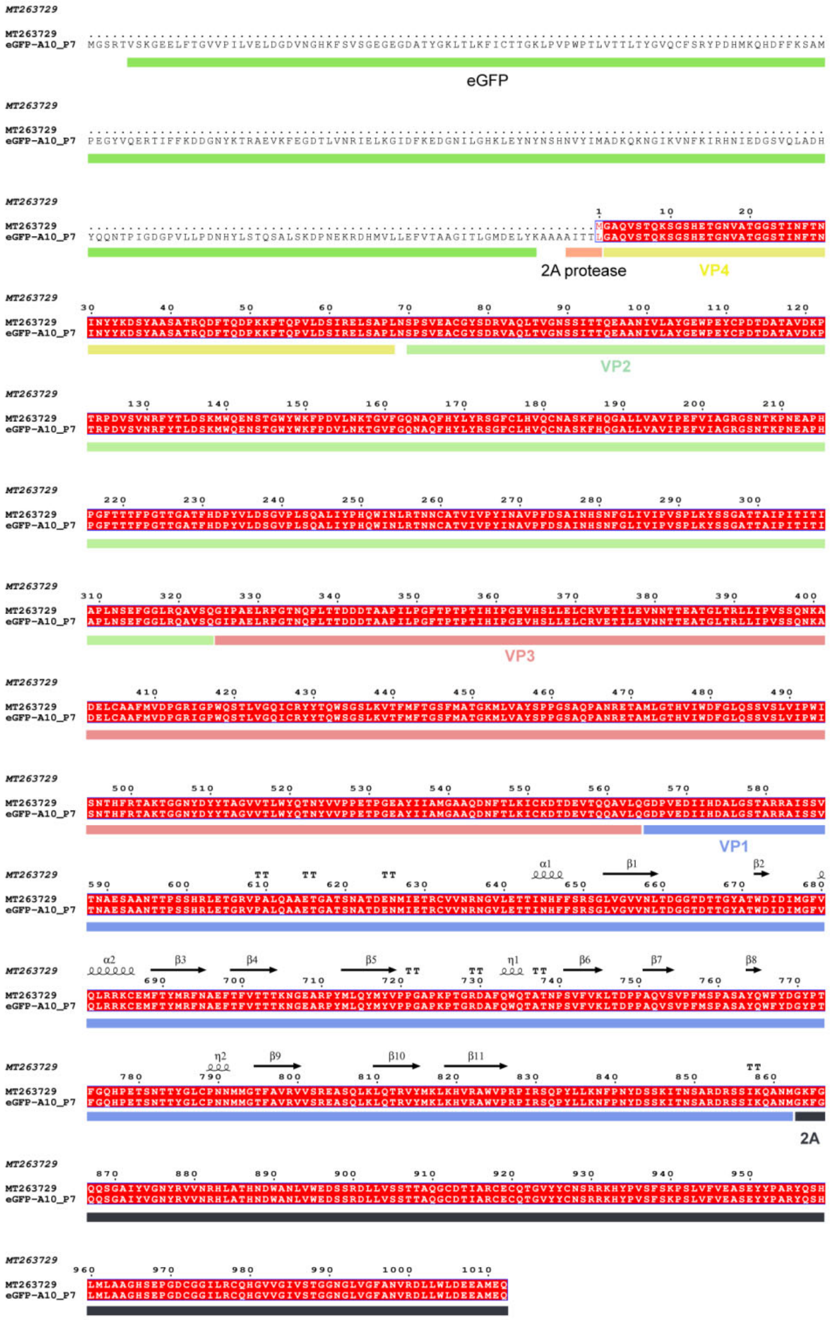

Figure S2. Sequence alignment of he P1 coding region of seventh-passage eGFP-A10 and parental CVA10. The reporter virus contains eGFP fused to the N-terminus of the CVA10 polyprotein via a 2A protease cleavage site (AITTL). The P1 region of the parental CVA10 strain (GenBank accession no. MT263729) was aligned with the corresponding region of eGFP-A10 P7 and visualized using ESPript. eGFP and VP4, VP2, VP3, VP1 and 2A are highlighted in green, yellow, green, red, blue, and black, respectively.

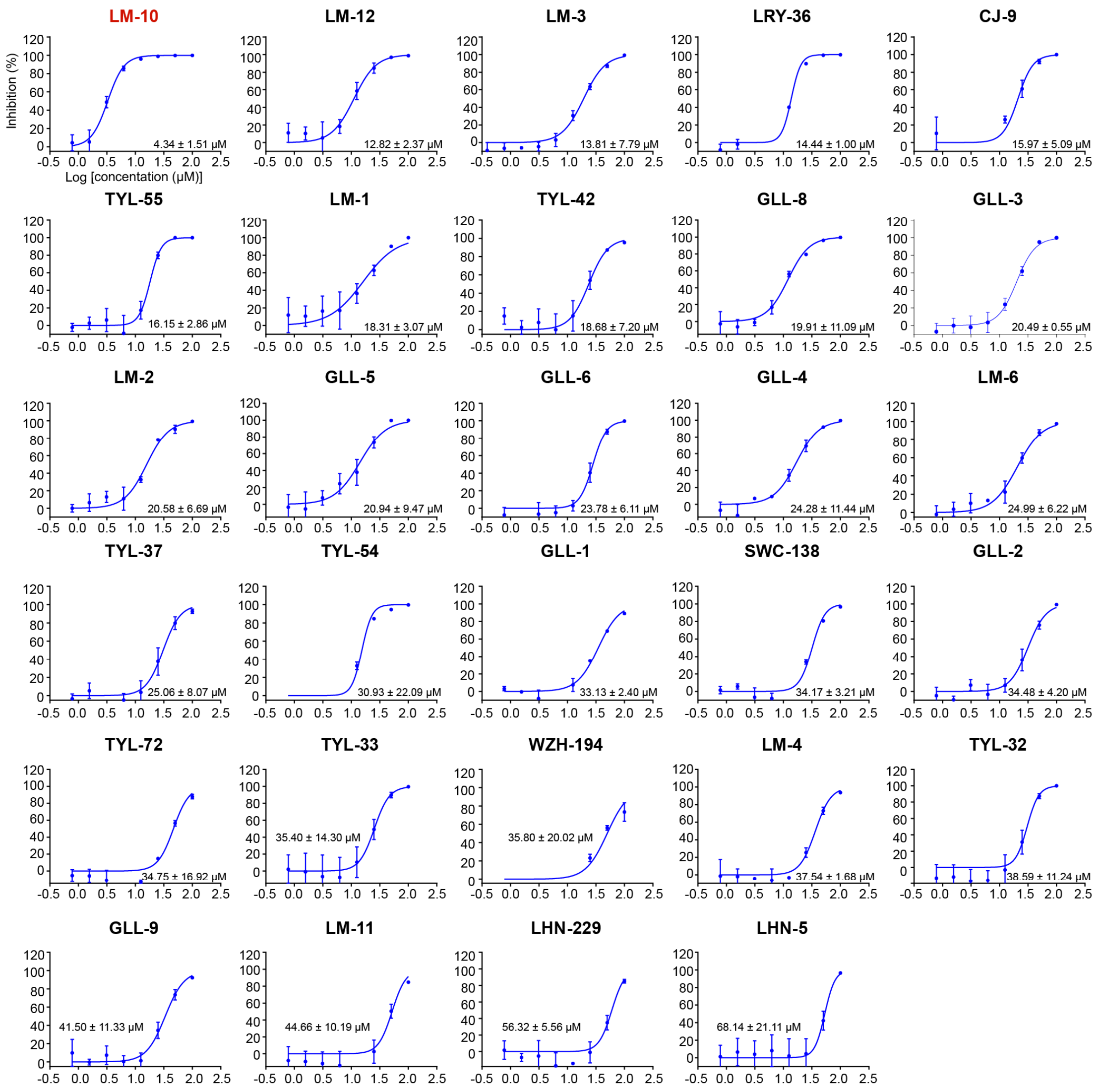

Figure S3. Dose-response inhibition of eGFP-A10 infection by the 29 candidate compounds. RD cells infected with the eGFP-A10 reporter virus were treated with serial dilutions of each compound, and inhibition of infection was quantified based on GFP reporter signal. Calculated IC_{50} values are indicated in each panel, and LM-10 is highlighted in red. All experiments were independently repeated at least three times.

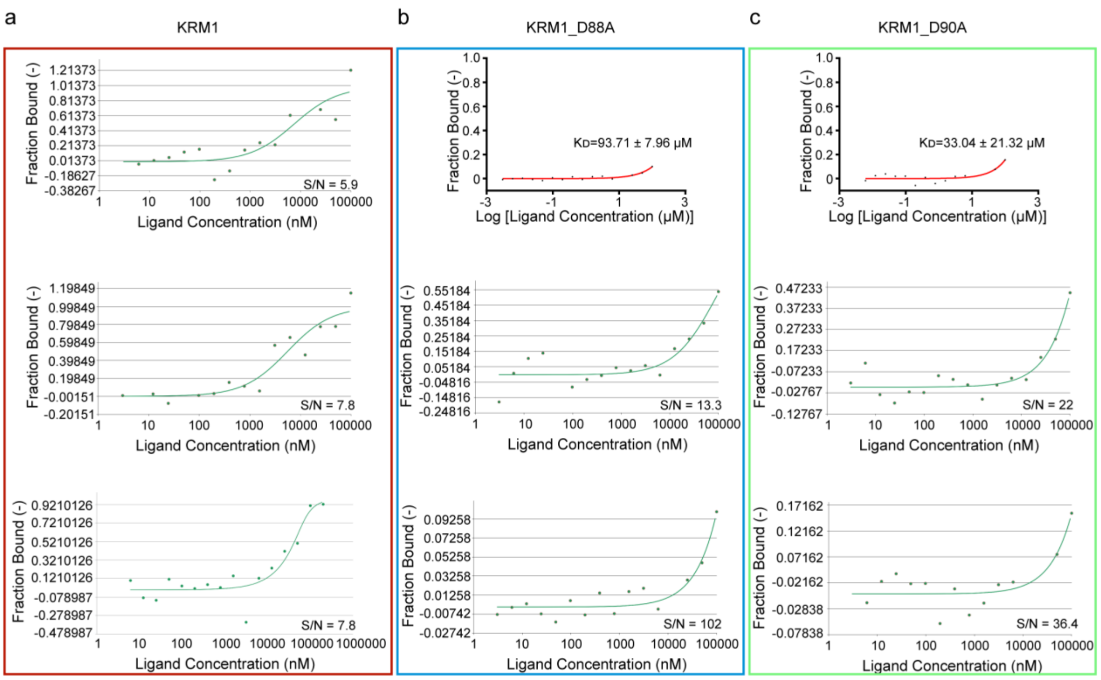

Figure S4. Microscale thermophoresis (MST) analysis of LM-10 binding to labeled KRM1 and its mutant proteins (D88A and D90A). (a) Raw MST traces for LM-10 binding to WT KRM1 from three independent measurements. (b, c) LM-10 was twofold serially diluted starting from 100 μM and mixed with labeled D88A (b) and D90A (c) at a final concentration of 100 nM, respectively. Data are representative of two independent experiments. A 10 mM phosphate buffer, pH 7.4 containing 137 mM NaCl, 2.7 mM KCl and 0.05% Tween-20 was used. Binding curves were generated using a Monolith instrument at low MST power and 20% excitation power, and the K_D value was determined by curve fitting, with an S/N > 5 for all measurements. Normalized fraction bound is plotted against ligand concentration.

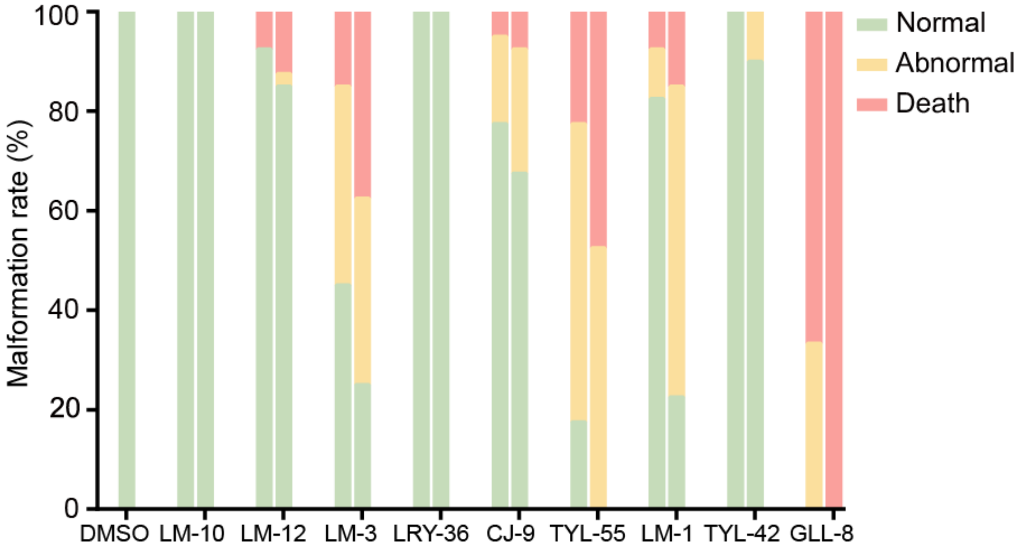

Figure S5. Developmental toxicity evaluation of test compounds (IC_{50} < 20 μM) in zebrafish embryos. Zebrafish embryos were exposed to the indicated compounds at concentrations of 5 or 20 μM from 6 h post-fertilization (hpf). Embryonic phenotypes were scored at 54 hpf and categorized as normal, abnormal, or death. The percentage of embryos in each category is shown. DMSO was used as the vehicle control. Data are representative of at least two independent experiments, with n ≥ 10 embryos per group.
